# Radiation Necrosis in Intracranial Lesions

**DOI:** 10.7759/cureus.7603

**Published:** 2020-04-09

**Authors:** Sean Munier, Elizabeth E Ginalis, Nitesh V Patel, Shabbar Danish, Simon Hanft

**Affiliations:** 1 Neurosurgery, Rutgers Robert Wood Johnson Medical School, New Brunswick, USA; 2 Neurosurgery, Rutgers Robert Wood Johnson Medical School, Piscataway, USA

**Keywords:** radiation necrosis, brain metastasis, brain tumor

## Abstract

Radiation necrosis (RN) is a challenging potential complication of cranial radiation therapy. Believed to result from a complex interplay of vascular, glial, and immunologic factors, the exact mechanism of RN remains unclear. Patients who develop RN typically have a history of treatment with stereotactic radiation surgery or some other form of radiation-based therapy. The time frame for its development is variable, but it most often occurs one to three years following radiation therapy. Reported treatment doses capable of inducing radiation necrosis are variable, with higher doses per fraction more likely to induce RN. Furthermore, RN remains a challenging diagnosis for clinicians to make, as its presentation is often nonspecific and imaging studies might not clearly differentiate RN from tumor recurrence or pseudoprogression. RN is initially managed with corticosteroids, followed by bevacizumab, surgical resection, or laser interstitial thermal therapy if symptoms persist. In this review, we examine the literature regarding pathophysiology, incidence, imaging characteristics, and management strategies for radiation necrosis.

## Introduction and background

Radiation necrosis (RN) is a focal structural anomaly that forms following cranial irradiation of cerebral neoplasms. The pathophysiology of RN remains poorly understood, though a number of mechanisms have been proposed and accepted as likely contributors. RN is a particularly challenging complication with a median onset of two years post-radiation, though the timeframe of presentation is variable and typically ranges from three months to 10 years post-radiotherapy [1-2]. The prognosis for patients with RN is typically poor, with one study citing a median survival time of 30 months following the development of necrosis [3]. Incidence is highest following high-dose local radiation, such as stereotactic radiosurgery (SRS) or brachytherapy, with reported rates after SRS ranging from 4%-19% [4-7]. Numerous factors play a role in the potential for the development of RN, including radiation dose, fraction size, and subsequent administration of chemotherapy [8]. The presentation of RN is highly variable but typically manifests with the reemergence of the initial symptoms related to the original tumor focus, with some cases presenting with new, unrelated neurologic symptoms. Based on symptomatology and imaging, RN may appear indistinguishable from tumor recurrence or pseudoprogression, thus making the diagnosis challenging [9]. Specifically, both RN and tumor recurrence may present with neurological deficits along with edema on T2 imaging, and contrast-enhanced studies may only show increased uptake secondary to disruption of the blood-brain barrier [10]. Tumor pseudoprogression is a well-recognized, self-limited post-radiation treatment effect defined as a transient increase in contrast enhancement followed by stability or regression. This may be seen in 20%-30% of patients following radiation and can also be difficult to distinguish from RN [11-12]. The lack of unique symptomatic or radiological findings in RN presents a diagnostic challenge for physicians, which may delay or prevent the initiation of an effective treatment modality. To date, multiple medical and surgical treatment options have been explored, with several novel options currently being investigated. In this review, we discuss proposed pathophysiologic mechanisms, incidence, diagnostic approach, and treatment options for patients with intracranial radiation necrosis.

## Review

Pathophysiology

**Figure 1:**
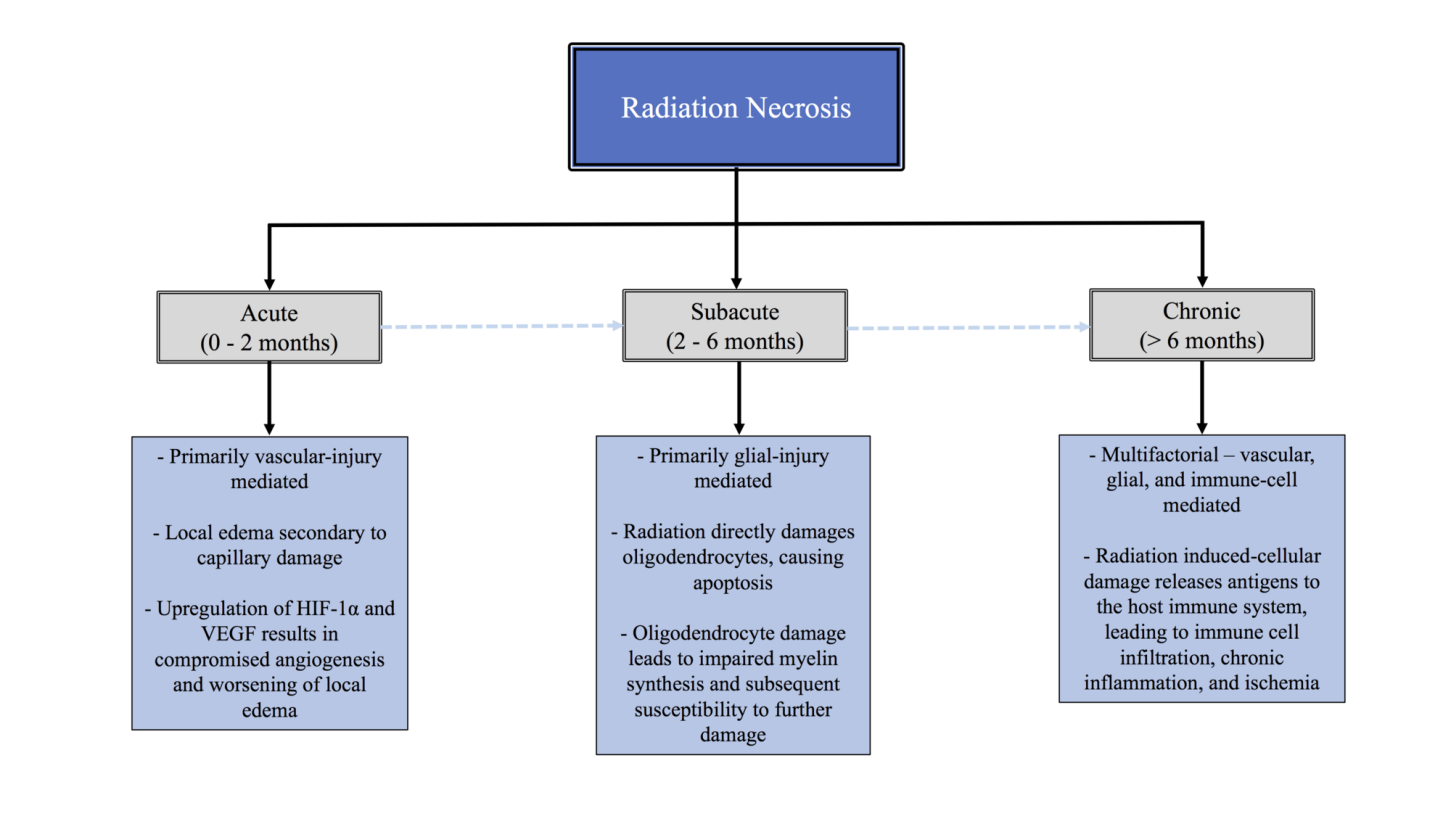
Tree diagram showing the stages of radiation necrosis with key features

The pathophysiology of RN remains poorly understood, though several proposed mechanisms are considered to be significant driving forces. The two major hypotheses to date are (1) the glial cell damage model, where RN is a result of direct injury to glial cells from radiation treatment and (2) the vascular injury model, in which RN arises from primary damage to blood vessels, which leads to subsequent brain parenchymal injury [10]. Both are believed to contribute to the natural evolution of RN. RN can generally be divided into three types: acute, subacute, and chronic [13]. The major features of these subtypes are summarized in Figure 1.

Acute RN

Acute RN is characterized by severe edema secondary to direct vascular injury and retraction of endothelial cells at the site of radiation, resulting in leakage of albumin into the interstitial fluid [14]. This breakdown of the blood-brain barrier is followed by a release of proinflammatory cytokines from local microglia, which further augment local edema and promote cellular death [15]. Specifically, due to radiation-induced hypoxia at the site of the irradiation, the upregulation of HIF-1α is believed to play a significant role in this setting. Previous reports have found that HIF-1α is upregulated in the perinecrotic area in radiation necrosis specimens [16]. HIF-1α is a well-known activator of vascular endothelial growth factor (VEGF) signaling, which results in the angiogenesis of leaky and fragile vessels resulting in local edema [9-10]. Typically, this edema will be radiologically evident immediately on post-therapeutic imaging.

Subacute RN

Subacute RN occurs weeks to months following radiation therapy and progresses from acute RN via a different mechanism. The progression of acute RN to subacute RN is believed to be secondary to radiation-induced oligodendrocyte dysfunction and apoptosis, which results in reduced production of myelin and subsequent local susceptibility for further cellular injury [17]. Along with endothelial cells and neural precursor cells, oligodendrocytes have been shown to be highly sensitive to radiation-induced damage by both p53 dependent and independent mechanisms [18-19]. Exposure to cytokines secondary to the vascular damage seen in acute RN exposes glial cells to the full effects of the inflammatory cascade, thereby promoting glial dysfunction. Subacute RN is clinically characterized by somnolence, fatigue, and, most notably, exacerbation of previous neurologic deficits related to the original tumor focus [14]. This type of RN has been shown to be responsive to steroids and may result in the cessation of symptom progression.

Chronic RN

In contrast to acute and subacute RN, the pathophysiology of chronic RN remains the most elusive and likely involves a combination of the factors contributing to acute and subacute RN.

In addition to the glial cell and vascular injury models, the immune-mediated model is a third mechanism proposed as a potential contributor to the development of delayed radiation necrosis. In the immune-mediated model, perivascular infiltration of T-cells along with upregulation of IL-6, IL-11, and TNFα and the production of reactive oxygen species collectively contribute to cellular injury [20]. Previous studies have investigated the nature of this phenomenon, demonstrating increased visibility of cancer cells to the immune system secondary to radiation exposure. Radiation therapy kills tumor cells, resulting in the release of cellular components, which can then act as antigens for the host immune system [21]. This can lead to a systemic immune response mediated via major histocompatibility complex (MHC) Class II and cluster of differentiation (CD) 4+ T cells not only restricted to the primary tumor but also to other metastatic sites as well [9]. Collectively, these findings are likely resultant from chronic inflammation induced by a complex interplay of the three proposed mechanisms, and determining which factor is predominant remains a challenge.

Incidence of radiation necrosis

The reported incidence of RN following radiation therapy has varied largely due to the heterogeneity of patient characteristics and radiation dose exposure in studied populations. Factors involved in the development of RN include radiation dose, fraction size, treatment duration, irradiated volume, tumor location, and subsequent administration of chemotherapy or radiosensitizers [5,8]. Additional challenges in determining the incidence of RN are due to the inability to capture all patients affected by RN, secondary to a lack of autopsies performed on patients potentially affected by RN and mortality from systemic disease progression in patients who may have subsequently gone on to develop RN. Furthermore, the presentation of RN is highly variable. Only certain patients may ultimately experience symptoms related to RN development. Specifically, one study found that in patients who developed RN, 41.3% of patients were symptomatic while the rest were asymptomatic [22]. This presents another obstacle to accurately determining the true RN incidence following radiation therapy. To date, RN has been shown to develop following a wide range of radiation modalities, including SRS, whole-brain radiation therapy, brachytherapy, and proton beam therapy [5,23]. Reported incidences of RN following treatment with SRS have typically ranged from 4%-18% [5-6,22,24]. Furthermore, RN can arise regardless of initial therapy indication, as it has been reported following the irradiation of metastatic lesions, primary tumors, and arteriovenous malformations [5]. To date, the strongest reported predictors for the development of RN appears to be radiation dose and irradiated volume, with V10 Gy and V12 Gy associated with high rates of RN [22]. In a series of 63 patients with a total of 173 brain metastases treated with SRS, RN occurred in up to 68.8% of patients treated with V10 Gy at a volume > 14.5 cm^3^ and V12 > 10.8 cm^3^ [24]. Conversely, no cases of RN were reported for V10 Gy < 0.68 cm^3^ or for V12 Gy < 0.5 cm^3^ [24]. A second study found that for fractionated radiation therapy with a fraction size < 2.5 Gy, the incidence of RN is 5% and 10% at biologically effective doses of 120 Gy and 150 Gy, respectively [25]. Again, while these studies provide some insight into identifying patients at risk for RN following varying doses of radiation, patient characteristic and treatment heterogeneity remain a challenge in accurately predicting the risk of developing RN.

Imaging characteristics

**Figure 2:**
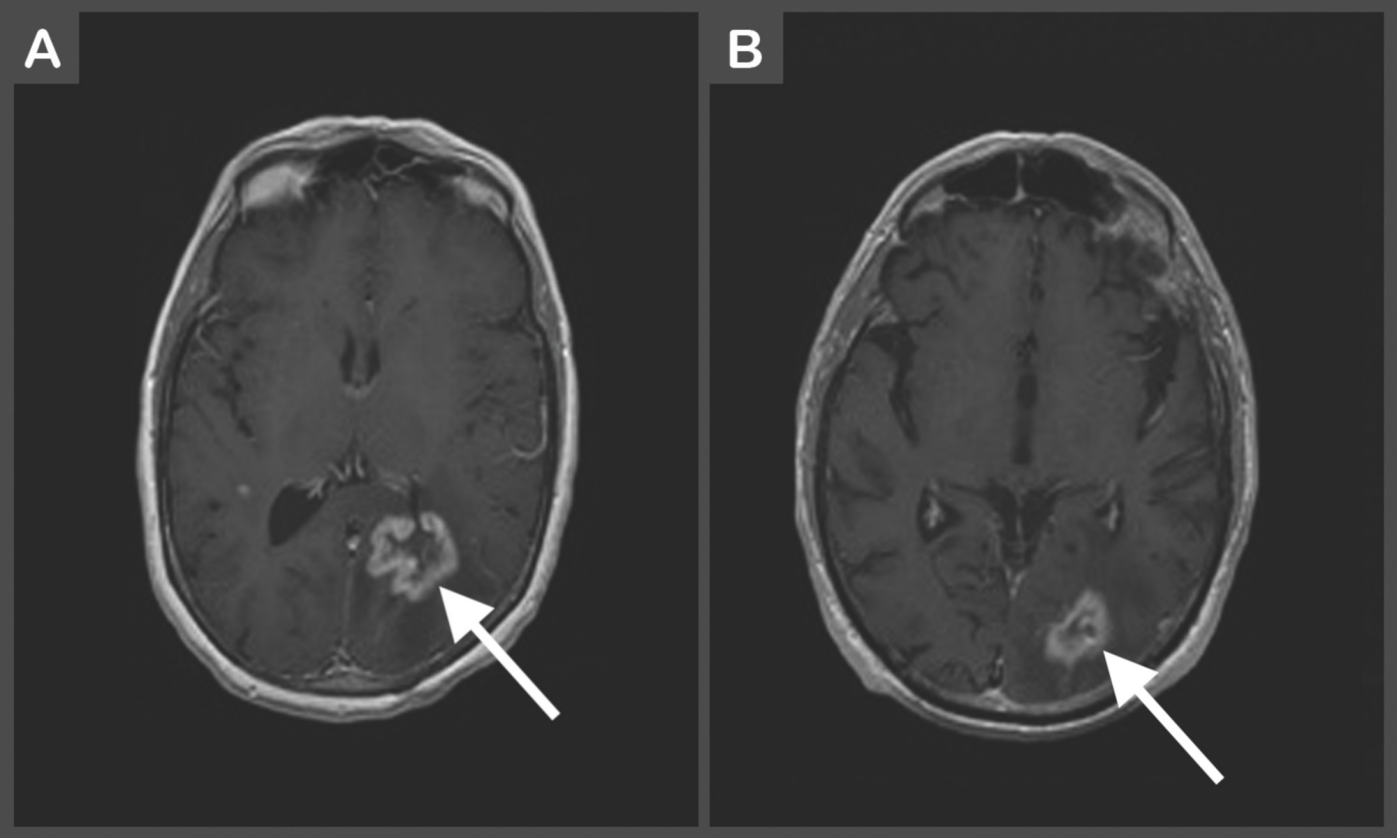
(A) Magnetic resonance contrast-enhanced T1-weighted image of left parietal lobe ring-enhancing lesion consistent with likely recurrent metastatic disease. (B) Magnetic resonance contrast-enhanced T1-weighted image showing enhancing lesion in the left occipital lobe suspected to be radiation-induced necrosis. This patient had undergone stereotactic radiosurgery with Gamma Knife two years prior. Gamma Knife: Elekta, Stockholm, Sweden

Perhaps the greatest obstacle in the management of RN is the initial diagnosis, as it can be difficult to differentiate RN from tumor recurrence and tumor pseudoprogression (Figure 2). Magnetic resonance imaging (MRI) alone is insufficient for diagnosis, as contrast enhancement can be seen in all three of these pathologies. As such, a multi-modality approach is essential. Radiographic diagnosis can be made using a combination of MR spectroscopy, diffusion-weighted imaging, diffusion tensor imaging, MR or computed tomography (CT) perfusion, single-photon emission CT (SPECT), and positron emission tomography (PET) [4]. The gold-standard for the diagnosis of RN is a biopsy. Histologic analysis of RN tissue samples shows calcification, fibrinoid deposition, vascular hyalinization, capillary collapse, and endothelial thickening as the long-term characteristics of RN [18,26]. However, biopsies are infrequently done due to the potential for complications and worsening of neurological status.

Historically, the Macdonald criteria were utilized as the standard for the evaluation of the treatment response of malignant gliomas. The criteria were based on utilizing two-dimensional measurements of enhancing tumors from CT scans, neurological status, and corticosteroid use (Table 1) [27]. More recently, the Response Assessment in Neuro-Oncology Working Group updated these criteria to include MRI (Table 2 and Table 3) [12]. Nonetheless, a notable limitation in both of these criteria is the inability to account for post-radiation effects. As such, it remains challenging to differentiate post-radiation treatment effects such as tumor pseudoprogression and RN from tumor recurrence by MRI alone. Consequently, a definitive diagnosis may require further imaging.

**Table 1:** Macdonald Criteria for Evaluating Treatment Response of Malignant Gliomas

Macdonald Criteria for Evaluating Treatment Response of Malignant Gliomas [12]	
Complete response	Requires all of the following: complete disappearance of all enhancing measurable and non-measurable disease for at least 4 weeks; no new lesions; no steroids; and stable or improved clinically
Partial response	Requires all of the following: ≥50% decrease compared with baseline in the sum of products of perpendicular diameters of all measurable enhancing lesions sustained for at least 4 weeks, no new lesions, stable or reduced steroid dose, and stable or improved clinically
Stable disease	Requires all of the following: does not qualify for complete response, partial response, or progression; stable clinically
Progression	Defined by any of the following: ≥25% increase in the sum of the products of perpendicular diameters of enhancing lesions, any new lesion, or clinical deterioration

**Table 2:** Response Assessment in Neuro-Oncology (RANO) Criteria *Progression occurs when any of the criteria is/are present.

Response Assessment in Neuro-Oncology (RANO) Criteria [12]				
	Complete Response	Partial Response	Stable Disease	Progressive Disease
T1-Gd+	None	≥50%	<50% decrease or <25% increase	≥25% increase*
T2/FLAIR	Stable or decrease	Stable or decrease	Stable or decrease	Increase*
New Lesion	None	None	None	Present*
Corticosteroids	None	Stable or decrease	Stable or decrease	NA
Clinical Status	Stable or increase	Stable or increase	Stable or increase	Decrease*
Requirement for Response	All	All	All	Any*

**Table 3:** Response Assessment in Neuro-Oncology (RANO) Criteria

Response Assessment in Neuro-Oncology (RANO) Criteria [12]	
Complete response	Requires all of the following: Disappearance of all enhancing disease (measurable and non-measurable sustained for at least 4 weeks; stable or improved on-enhancing FLAIR/T2 lesions; no new lesions; no steroids (physiological replacement disease allowed); clinically stable or improved
Partial response	Requires all of the following: ≥50% decrease of all measurable enhancing lesions sustained for at least 4 weeks; no progression of non-measurable disease; stable or improved non-enhancing FLAIR/T2 lesions; no new lesions; stable or reduced steroids (compared to baseline); clinically stable or improved
Stable disease	Requires all of the following: does not qualify for complete response, partial response, or progression; stable clinically
Progression	Defined by any of the following: ≥25% increase in enhancing lesions despite stable or increasing steroid dose; significant increase in non-enhancing T2/FLAIR lesions, not attributable to other non-tumor causes; any new lesions; clinical deterioration (not attributable to other non-tumor causes and not due to steroid decrease)

Typical non-specific findings of RN on MRI include necrotic foci, contrast enhancement, and perilesional edema [28]. However, these findings are not unique to any one pathology and thus render them ineffective criteria for the diagnosis of RN. One previous study aimed to define specific MRI features that may assist in distinguishing RN from tumor recurrence and identified the following: arteriovenous shunting, gyriform lesion/edema distribution, perilesional edema, and cyst formations. However, each feature was found to have poor sensitivity [29]. Additionally, the authors defined a “lesion quotient” based on the following: in nodules with definable borders as observed on T2-weighted sequence, the maximal cross-sectional area was calculated and compared with the area encompassed by the contrast enhancement on the T1-weighted post-gadolinium sequence on a comparable axial section [29]. A lesion quotient of 0.6 or greater was found to have a negative predictive value of 88% for RN. This study, though based on a single radiologist’s findings, provides a promising mechanism for distinguishing RN from recurrent tumors.

Other mechanisms for differentiation between tumor progression and radiation effects have been reported. One such mechanism is T1/T2 matching. A T1/T2 match occurs when the border of a nodule or lesion wall appears hypointense on the T2-weighted scans and matches or partially matches the border on the T1-weighted enhanced images [30]. Failure to meet these criteria is termed a T1/T2 mismatch. Under this scheme, cases with T1/T2 matching were found to be highly correlated with tumor recurrence while mismatch cases were more likely to be associated with RN [30]. This method had a sensitivity of 83.3% and a specificity of 91.1% for identifying necrosis. Since it has no technical measurements and can be performed quickly, T1/T2 matching may be a more practical approach to identifying necrosis as compared to the lesion quotient previously described.

Several other imaging modalities have been identified as useful in differentiation between RN, pseudoprogression, and tumor recurrence. Specifically, MR perfusion, MR spectroscopy, 6-[(18)F]-fluoro-L-3,4-dihydroxyphenylalanine (F-DOPA)/FDG PET, 1-methyl-(11)C-methionine ((11)C-methionine ((11)C-MET), and SPECT scan have been shown to be viable options in differentiating RN from other pathologies [31]. The sensitivity and specificity of MR perfusion MRI and F-DOPA PET have been reported to be 86.7% and 68.2% and 90.0% and 92.3%, respectively [32]. A SPECT scan has been shown to have the highest specificity at 97.8% and a sensitivity of 87.6% for differentiating tumor progression and radiation necrosis [33].

Pathologic considerations

When attempting to differentiate between tumor recurrence and radiation necrosis, initial indication for radiation is an important factor to consider. Specifically, in the setting of new enhancement on follow-up imaging after radiation, whether the patient was originally treated for a primary glioma versus metastatic disease may assist in guiding management. This is because the control of metastatic lesions by radiation is higher compared with glioblastoma multiforme or high-grade gliomas [34]. Therefore, there is a higher baseline likelihood that post-imaging changes seen after radiation of metastatic lesions can be attributed to radiation effects whereas true recurrence is more likely with gliomas. In one study evaluating the treatment of brain metastases with radiosurgery, 23 patients underwent surgery for pathologic diagnosis after having suspicious findings on post-treatment MRI. Of these, 22 of 23 demonstrated radiation-induced change without any evidence of active tumor on pathology [35]. This is in contrast to a study that histologically evaluated 27 patients with primary gliomas treated with radiation and newfound enhancement on MRI. Though all patients were found to have some degree of residual tumor, 15 patients were found to have predominant tumor recurrence and 12 patients predominantly had RN [20]. This idea of mixed RN and tumor recurrence raises another challenging question regarding the treatment approach. To date, there is no clearly defined criteria for determining whether the primary underlying pathology should be treated. A better understanding of this complex question is critical, as it would assist in decision-making when considering conservative treatment versus aggressive intervention. As such, further studies will be needed going forward to better guide management in the setting of mixed pathology.

Successfully differentiating RN from tumor progression using imaging was also impacted by the initial brain lesion. In one systematic review, RN could be diagnosed by any radiological imaging, including gadolinium-enhanced MRI, in patients with metastatic brain tumors, whereas the diagnosis was challenging for patients with gliomas. In patients with gliomas, combined imaging that includes metabolic and blood flow methods enhanced the diagnostic accuracy for differentiating RN from tumor progression in this study [36]. Together, these results suggest that the patient’s initial lesion should be considered during the evaluation of RN in order to guide the selection of imaging modalities for diagnosis.

Treatment modalities

RN can be managed either medically or, in select cases, surgically. Currently, medical management is the initial approach to patients with symptomatic RN. Initial treatment is with corticosteroids to decrease cerebral edema. Steroids are typically effective as a short-term solution to RN, as they often succeed in reducing local edema related to the RN. However, this treatment has significant adverse effects, including anxiety, depression, gastrointestinal disturbances, hypertension, and swelling of the hands, feet, and face [37]. For patients who remain symptomatic with corticosteroid therapy or whose symptoms return during corticosteroid tapering, a course of bevacizumab is recommended. Bevacizumab is an anti-VEGF monoclonal antibody that appears to be a promising treatment option for patients with RN. As previously discussed, VEGF signaling appears to play a prominent role in the development of RN. Gonzalez et al. were the first to describe the efficacy of bevacizumab as an additional chemotherapeutic agent in patients with recurrent malignant gliomas involving RN, finding it was effective in reducing symptomology [38]. Since then, several studies have further investigated its efficacy as a treatment modality with promising results [3]. Most recently, a randomized, double-blind clinical trial of 112 patients with radiation-induced brain necrosis compared two months of bevacizumab monotherapy to standard corticosteroid treatment and found that 65.5% of patients showed symptomatic improvement in the bevacizumab group compared with 31.5% in the corticosteroid group. Furthermore, patients in the bevacizumab group showed a 25.5% reduction in lesion volume on T1-weighted imaging as compared with only 5.0% in the corticosteroid group [3]. These findings, though within a limited population, provide promising evidence of bevacizumab as an effective option for patients with RN. Although bevacizumab shows good efficacy for improving symptoms, RN recurrence after bevacizumab discontinuation has been described [39]. As such, additional studies are needed to evaluate bevacizumab as a treatment option.

Lastly, surgical resection of the necrotic tissue is considered only for refractory cases who have failed conservative treatment or those with contraindications to bevacizumab. Surgical intervention may improve symptoms by rapidly decreasing mass effect and brain edema. However, surgical excision carries substantial risk for worsening of patient neurological status, and, furthermore, not all lesions may be accessible via open resection depending on location.

Laser ablation

**Figure 3:**
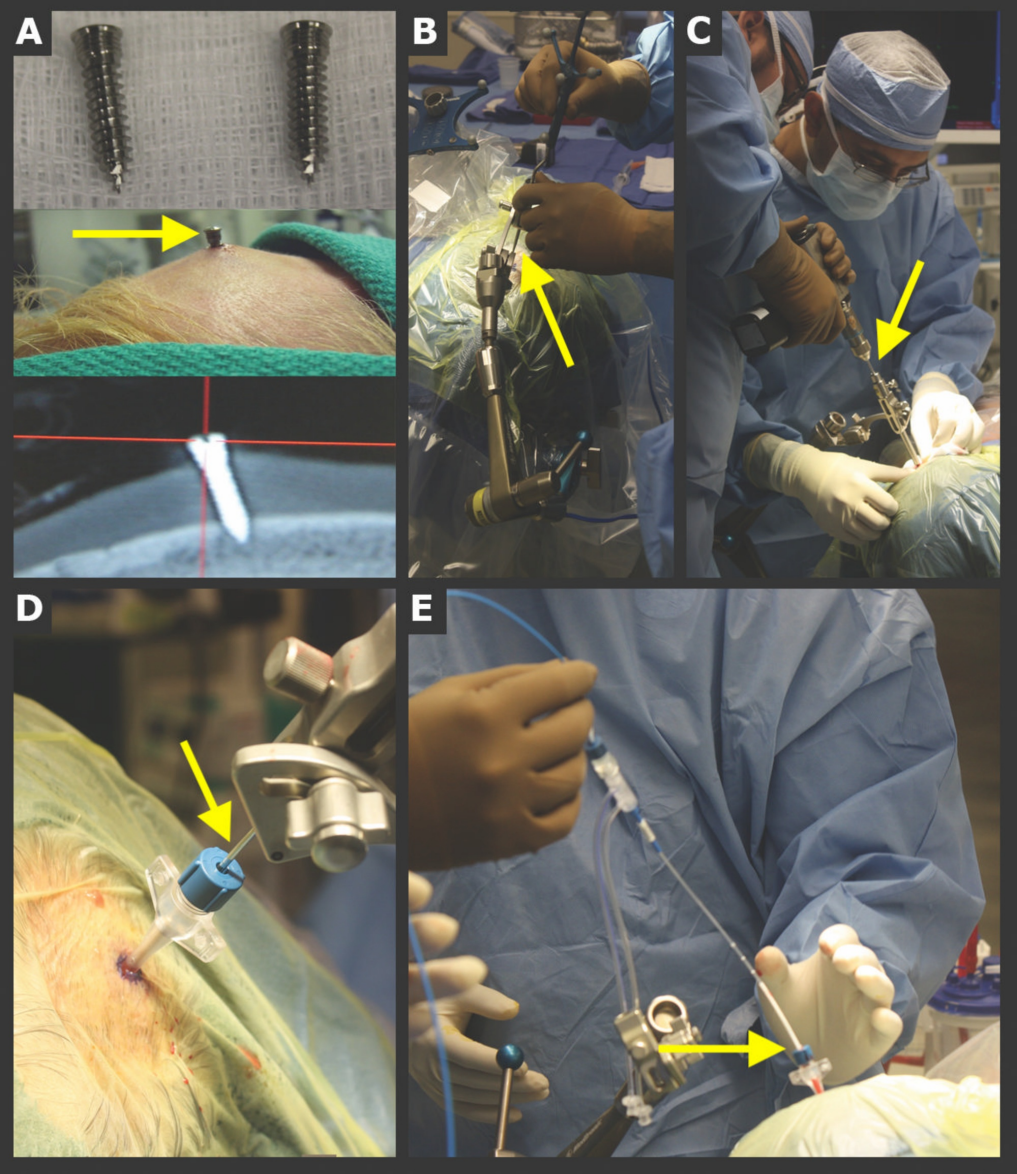
Implanted skull fiducial system: general steps for laser catheter placement (A) The pins (screws) shown are placed circumferentially in the skull, typically totaling ∼6 pins. The pinheads are used as registration points. (B) The precision aiming device (PAD) is aligned with the planned trajectory using the stereotactic handheld probe, and the PAD is then locked. (C) An automated drill is used to drill a twist drill hole, and a reducing cannula followed by a rigid stylet is passed to ensure the completeness of the twist drill hole. (D) Next, the bone anchor is placed and the dura is perforated. (E) The laser catheter is then passed to the planned depth (image used with permission from Patel et al. [43]).

**Figure 4:**
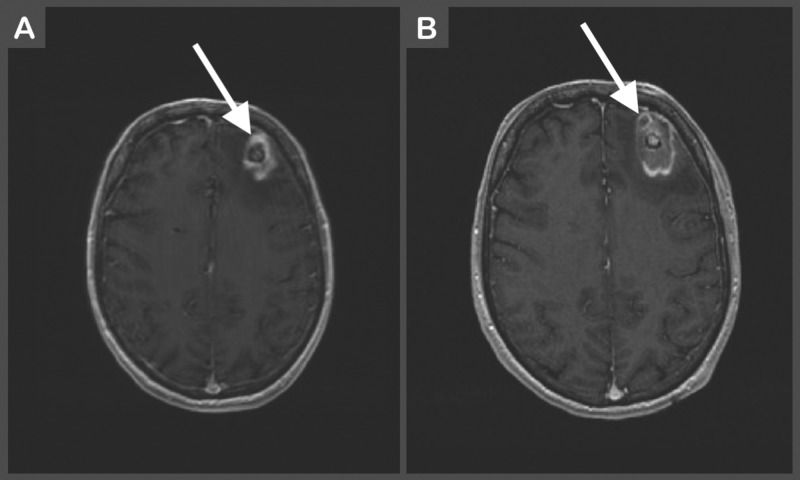
(A) Preoperative contrast-enhanced T1-weighted image of left frontal mass with surrounding vasogenic edema likely secondary to radiation necrosis. (B) Postoperative LITT contrast-enhanced T1-weighted image showing mixed-signal core intensity with thin peripheral enhancement consistent with expected complex hematoma. LITT: laser-induced thermal therapy

Due to the risks associated with surgical debulking, alternative, minimally invasive techniques have been investigated. Laser-induced thermal therapy (LITT) has become a viable and effective treatment option for RN with obvious benefits due to its minimally-invasive nature (Figure 3). LITT’s efficacy has previously been demonstrated in a study by Patel et al. where 37 patients with recurrent metastasis or radiation necrosis were treated via intracranial laser ablation (Figure 4) [40]. Total operative time and ablation duration were 2.8 ± 0.6 hours and 8.7 ± 8.1 mins, respectively. Postoperative complications included neurological worsening (n = 7), hemorrhage (n = 1), edema (n = 1), infection (n = 1), and thermal injury to the pituitary leading to secondary complications (n = 1) [41]. Follow-up metrics, such as overall survival and progression-free survival, were not reported in this study. Nonetheless, it provides evidence that LITT is a relatively safe and effective treatment modality for patients with RN. A second study by Rao et al. also evaluated the use of LITT as a treatment option for patients with RN. They performed a case series of 16 patients who underwent MR-guided LITT for RN or metastatic recurrence. Local control was achieved in 75.8% of patients. Median progression-free survival was found to be 37 weeks [42]. Again, though performed within a small population, this study also provides evidence of LITT as an effective treatment modality for RN.

An important consideration for patient selection for LITT versus surgical resection is the degree of mass effect. Rammo et al. studied the safety of LITT for RN and identified post-ablation edema as a side effect of LITT that resulted in a permanent neurological decline in one patient and transient focal neurological deficits in three other patients [4]. This indicated that post-ablation edema is generally tolerated in patients without significant mass effect prior to LITT. However, surgical resection may be preferred over LITT in patients with significant mass effect prior to intervention if the lesion location and patient co-morbidity are favorable for surgery.

Overall, LITT appears to be a promising and safe treatment for RN, especially in patients who are refractory to medical treatment. It is a minimally invasive alternative to surgical resection. Future studies are needed to continue defining the role of LITT in RN management. Particularly, it remains to be seen if LITT is a comparable or even superior treatment as compared to medical therapy, which is the current first-line therapy for RN.

Novel therapies

Several novel treatment options have also been investigated for RN. Amongst them are hyperbaric oxygen therapy (HBOT), oral vitamin E with pentoxifylline, and anticoagulation with heparin and warfarin [44-45]. HBOT is an interesting approach to RN therapy. Currently, there are no double-blind, placebo-controlled trials to prove its efficacy, but several case studies and prospective studies have demonstrated some benefit [46]. Oxygen is delivered at 20-24 atmospheres for 20-30 sessions, each session lasting 90-120 minutes [47]. The basis of the therapy is to increase oxygen concentration in ischemic areas and promote tissue healing via improved angiogenesis, thereby improving tissue perfusion and halting disease progression [4,44,48]. Whereas anti-VEGF therapy aims to prevent impaired angiogenesis from occurring altogether due to radiation-induced hypoxia, HBOT aims to improve VEGF-based angiogenesis by augmenting local oxygen delivery. Furthermore, the therapy is believed to reduce tissue edema and increase collagen synthesis via fibroblasts, which is critical in restoring damaged tissue [44]. These thoughts, while speculative, are intuitive in the context of RN pathophysiology, which is believed to be largely related to perfusion deficits secondary to chronic inflammation and vascular damage. Still, despite promising results, there are numerous pitfalls to HBOT that make it a less appealing option. Specifically, it is expensive, time-consuming, and not universally available. Additionally, it has a significant toxicity profile, including cataract enhancement, ear barotrauma, pneumothorax formation, hypoglycemia in diabetic patients, and oxygen-associated seizures [44].

Combination vitamin E and pentoxifylline is another interesting treatment modality in the management of radiation-induced fibrosis. Because the production of reactive oxygen species is believed to play a role in the pathophysiology of RN, the use of antioxidants such as vitamin E and pentoxifylline have been investigated. Pentoxifylline additionally works by improving circulation via increased blood cell deformity and decreased viscosity [7]. Most notably, a randomized, placebo-controlled trial of combined vitamin E and pentoxifylline found that, at six months, combination therapy resulted in a 53% reduction in the radiation-induced fibrosis surface area [49]. This is a more promising therapy compared with HBOT, as these two drugs are commercially available and well-tolerated.

Lastly, therapeutic anticoagulation therapy is another alternative approach that has been reported in a few small case studies but is not currently recommended in standard management [7,39]. The use of anticoagulation with warfarin and heparin has been suggested as RN pathophysiology involves vascular damage resulting in vessel thrombosis and occlusion. In one study by Glantz et al., five out of eight patients with biopsy-confirmed RN showed some clinical improvement with the use of heparin followed by warfarin for three to six months. However, one patient’s symptoms recurred after the anticoagulation was discontinued [45]. In a more recent study by Happold et al., two of three patients with cerebral RN lesions reported minor improvement of clinical symptoms [50]. Overall, anticoagulation therapy seems to have only a modest effect on improving systems in patients with RN.

## Conclusions

Radiation necrosis is a devastating and challenging complication of radiation therapy. The pathophysiology of RN is complex and multifactorial. An accurate and timely diagnosis remains a significant hurdle for practitioners, though advanced imaging techniques have helped ameliorate this obstacle. The current management of RN includes initial treatment with corticosteroids followed by bevacizumab in patients who remain symptomatic despite steroid therapy. Surgical resection can be considered in medically refractory cases. Beyond standard management, promising treatment modalities have emerged, including LITT and combined vitamin E with pentoxifylline, affording physicians several options when managing this diagnosis. Further studies will be necessary to identify additional treatment approaches and elucidate the precise nature of RN development.
